# Knowledge, Attitude and Practice Regarding Organ Donation among Indian Dental Students

**Published:** 2016-02-01

**Authors:** K. Chakradhar, D. Doshi, B. Srikanth Reddy, S. Kulkarni, M. Padma Reddy, S. Sruthi Reddy

**Affiliations:** Department of Public Health Dentistry, Panineeya Institute of Dental Sciences and Hospital, Hyderabad, India

**Keywords:** Tissue and organ procurement, Directed tissue donation, Organ transplantation, Students, dental, India, Health personnel, Knowledge, Attitude

## Abstract

**Background:**

Of the overall 9.5 million deaths annually in India, nearly 100,000 are due to organ failure. To save and extend lives, organ donation and organ transplantation have become the only hope. Health care professionals (HCPs) are a key element in facilitating cadaveric organ donation process.

**Objective:**

To assess and compare the knowledge, attitude, and practice regarding organ donation among undergraduate dental students.

**Methods:**

A cross-sectional study was conducted among 298 undergraduate dental students of the Panineeya Institute of Dental Sciences and Hospital, Hyderabad, India. A 27-item self-administered questionnaire, which assessed the levels of knowledge (Q1–13), positive attitude (Q14–24) and practice habits (Q25–27) regarding organ donation with dichotomous scale (Yes/No).

**Results:**

As compared to males, females reported better mean±SD scores in knowledge (8.22±1.51) and practice (0.91±0.8); higher mean±SD attitude scores (8.55±1.56) were reported among males (p<0.001). While second year dental students had higher scores for their knowledge (8.55±1.56) and practice (1.02±0.44) compared to other year of training, third year students showed a significant higher mean attitude score (1.73±1.17) (p=0.02). Hindus and Muslims scored significantly lower mean knowledge, attitude and practice habits compared to others (Christians, Jains and Athesists) (p<0.001). There was a positive correlation between mean knowledge, attitude, and practice habits.

**Conclusion:**

There are an average level of knowledge and low levels of positive attitude and practice habits among studied dental students towards organ donation and transplantation.

## INTRODUCTION

Of a total of 9.5 million deaths annually in India, nearly 100,000 are due to organ failure [1]. To save and extend lives, organ donation and organ transplantation have become the only hope [2, 3].

Since the first transplantation performed in 1954, the ability to enhance and extend life using this procedure has advanced from the experimental stages to that of standard practice [4]. Transplantation is defined as the transfer (engraftment) of human cells, tissues or organs from a donor to recipient with an aim of restoring function(s) in the body [5].

Among 100,000 of people died each year are believed to be potential donors; however, only less than 200 actually become donors [1]. The Government of Andhra Pradesh has initiated the deceased donor program under the banner of “Jeevandaan,” and has been facilitating and taking care of organ distribution, however, it could not retrieve more than 246 organs by 2013. Therefore, the shortage of organ donations had been a major limiting factor in transplant programs, inspite of improvement in graft and patient survival rate [6].

At any point of time, 8–10 brain-dead patients have been recorded in various ICUs in our country. The conversion of these brain-dead patients into donors would end the long waitlist [1]. To increase the number of potential and actual donors, artificial and bio-artificial organs, “non-heart-beating donors” and sub-optimal donors have been used, but worldwide, more than 20% of patients on waiting lists (mainly for liver and heart) die every year because of the shortage of donor organs.

The issue of organ donation is complex and multifactorial involving ethical, legal, medical, organizational, and societal factors [7]. Countries around the world have reported that people’s attitudes toward organ donation are influenced by factors such as knowledge, education, and religion [8].

To achieve cadaver organ donation, it is necessary to act at two levels: the general public and health care workers [9]. Health care professionals (HCPs) form a key element of the cadaveric organ donation process [8]. The attitudes of health care specialists, trainees, and nurses is of great importance. Insufficient knowledge and failure to identify possible donors are considered important contributing factors responsible for the shortage of available organs.

Education programs have recently been suggested as a new approach in solving the organ shortage [10]. The general population and the student population in particular, need to be educated about transplantation and the need to accept the commitment to donate organs. The undergraduate medical and dental curriculum should provide students with basic information on procedures and ethical issues concerning organ transplantation and donation, so that future doctors can become informed advocates [9].

The literature review revealed that no research has been conducted on knowledge, attitude and practice regarding organ donation among dental students. Therefore, the present study was designed to assess and compare the knowledge, attitude and practice regarding organ donation among dental students, based on gender, year of study and religion.

## MATERIALS AND METHODS

A cross-sectional questionnaire-based study was conducted among undergraduate dental students of Panineeya Institute of Dental Sciences and Hospital, Hyderabad, India. The study sample comprised of first-, second-, third- and fourth-year dental students. Anonymity and confidentiality of respondents were maintained and participation was voluntary. Ethical approval for this study was obtained from the Ethical Committee of the Institutional Review Board (PMVIDS/PHD/0025/2014).

A 27-item self-administered questionnaire was developed based on previous studies [7, 11, 12] comprising of four sections. The first section of the questionnaire gathered the demographic details from the students, which included age, gender, year of study and religion. The second, third and fourth sections assessed the levels of knowledge (Q1–13), positive attitude (Q14–24) and practice habits (Q25–27) regarding organ donation, respectively. The responses were recorded on a dichotomous scale (Yes/No). For each “Yes” response it was scored ‘1’ and for each “No” response ‘0.’ Reverse scoring was done for the questions (Q6, 9, 10, 21 and 23) where the correct responses were “No.” The total scores obtained were summed up. The higher scores indicated better knowledge, more positive attitude and good practice habits regarding organ donation.

The questionnaire was distributed to undergraduate dental students during lecture hours in the classroom. The participants were instructed not to discuss the questions among themselves. Only completed questionnaires were utilized for the study. Data were entered in an MS Excel^®^ sheet and analyzed by SPSS^®^ for Windows^®^ ver 20. *Student’s t *test and one-way ANOVA were used for comparing means of normally distributed continuous variables. Correlation between knowledge, attitude and practice scores was calculated by Pearson’s correlation coefficient. A p value <0.05 was considered statistically significant.

## RESULTS

Out of 345 participants, 298 undergraduate dental students completed the questionnaire and were included in this study—a response rate of 86.3%. The study population included 238 (79.9%) females and 60 (20.2%) males with a mean±SD age of 19.6±1.7 years. Most of the study participants belonged to first year (n=84, 28.2%), followed by third year (n=75, 25.2%), fourth year (n=70, 23.5%), and second year (n=69, 23.1%). When religion was considered, the majority of participants were Hindus (n=219, 73.5%), with only a small percentage being Muslims (n=46, 15.4%) and other religion constituting Christians, Jains, and Atheists (n=33, 11.1%).

Comparison of the correct knowledge responses, based on gender revealed that though females had a better knowledge as compared to males, significant difference was noted only for questions 1, 3 and 12 (p<0.05). When year of study was considered, majority of first-year students had correctly responded to knowledge questions (Q1–13) compared to other years. However, a significant difference was observed for questions 1–4 and Q6 (p<0.05). Furthermore, for questions 9 and 10, fourth-year students had a significantly higher knowledge; likewise for questions 11 and 12, third-year students had better knowledge. Hindus had more correct knowledge with regard to organ donation with significant (p<0.05) difference for most of the questions except Q2–3, and Q9–10 (Table 1).

**Table 1:** Questions asked for the assessment of knowledge, attitude, and practice responses among the study population stratified based on gender, year of study, and religion

Question		p value		
		Gender	Year of study	Religion
1	Have you heard of the term “organ donation?”	<0.001	<0.001	<0.001
2	Have you heard of the term “organ transplantation?”	0.80	<0.001	0.51
3	Are you aware of “transplantation of human organs act?”	0.01	<0.001	0.42
4	Do you know where to obtain organ donation cards?	0.86	<0.001	0.04
5	Can a brain-dead patient’s organs be donated?	0.76	0.82	0.01
6*	Will certified brain-dead registered organ donor be immediately disconnected from ventilation support?	0.29	0.02	0.04
7	Can parents/guardians make substitute decision making for mentally disabled persons in the regard of organ donation?	0.10	0.64	0.05
8	Donor’s and recipient’s blood group MUST be matched?	0.08	0.49	<0.001
9*	Donor’s human leukocytes antigen MUST be identical to that of the recipient for any organ transplantation?	0.40	<0.001	0.27
10*	Hepatitis B and C carriers can donate all of their solid organs except the liver organs ?	0.82	0.05	0.09
11	Malignancy is always a contraindication to cadaveric organ donation?	0.58	<0.001	<0.001
12	Increased risk of opportunistic infections is a common complication to all transplantations?	0.04	0.05	0.01
13	Organ transplant recipients are more prone to developing of cancer after transplantation?	0.56	0.25	<0.001
14	Do you support organ donation?	<0.001	0.82	<0.001
15	Do you feel comfortable to think or talk about organ donation?	0.01	0.13	0.04
16	Do you agree to donate organs when you die?	<0.001	0.14	<0.001
17	Do you agree to donate your family member’s organs?	0.16	0.05	<0.001
18	Does your family agree with organ donation?	<0.001	0.06	<0.001
19	Do you think donating one’s organ adds meaning to one’s life?	0.01	0.95	<0.001
20	Does your religion agree with organ donation or transplantation?	0.02	0.28	<0.001
21*	Do you have belief that your body should be kept intact after death?	<0.001	0.02	<0.001
22*	Do you have fear that your body will be disfigured, if you donate organs?	0.29	0.02	0.03
23*	Do you think there will be premature termination of medical treatment for registered organ donors?	0.63	<0.001	<0.001
24	Do you think live organ donation is better than cadaveric organ donation in solving shortage?	0.24	<0.001	<0.001
25	Have you pledged/signed to donate an organ?	0.05	0.10	<0.001
26	Have you ever donated an organ?	<0.001	0.04	0.30
27	Did you ever receive an organ for transplantation?	0.01	0.18	<0.001

* The correct response for these questions is “No;” for all other questions the correct response is “Yes”

Comparison of positive attitude responses based on gender revealed that females had a higher positive attitude as compared to males; the difference was significant (p<0.05) for most of the questions (Q14–16, and Q18–21). Based on the year of study, a significant difference was found for questions 17, 21 and 22 among the first-year participants followed by Q23 for the final year and Q24 for the third-year students. Hindus reported a more positive attitude than Muslims, Christians, Jains and Atheists (others) with significant (p<0.05) difference for all attitude questions Q14–24 (Table 1).

When good practice responses regarding organ donation were measured and compared according to gender, a significantly more positive response was shown by females for Q25 and males for Q26 and Q27. Furthermore, second-year students reported a significantly higher response for Q26 when compared to other students. Based on the religion, Hindus revealed a higher response for Q25 and Q27, compared to other religions (p<0.05) (Table 1).

When levels of correct knowledge were considered, the majority of the participants (n=195, 65.4%) had average knowledge (50%–75%); it followed by 68 (22.8%) with high levels of knowledge (>75%) and 35 (11.74%) with low levels of knowledge (<50%). 

Table 2 depicts that based on gender and religion, females (n=236, 99.2%), and Hindus (n=219, 100%) had a significantly (p<0.001) lower levels of positive attitude. Comparison based on year of study did not reveal any significant difference (p=0.28).

**Table 2:** Comparison of the levels of correct knowledge, attitude, and practice based on gender, year of study, and religion

Variables	Knowledge					Attitude					Practice			
	Low n (%)	Average n (%)	High n (%)	p value		Low n (%)	Average n (%)	High n (%)	p value		Low n (%)	Average n (%)	High n (%)	p value
Gender														
Males	10 (17)	39 (65)	11 (18)	0.33		56 (93)	4 (7)	0 (0)	<0.001		56 (93)	4 (6.7)	0 (0)	0.24
Females	25 (10.5)	156 (66)	57 (24)			236 (99.2)	2 (0.8)	0 (0)			230 (96.6)	8 (3.4)	0 (0)	
Year of study														
First Year	11 (13)	56 (67)	17 (20)	0.08		83 (99)	1 (1)	0 (0)	0.28		82 (98)	2 (2)	0 (0)	0.81
Second Year	3 (4)	42 (61)	24 (35)			66 (96)	3 (4)	0 (0)			66 (96)	3 (4)	0 (0)	
Third Year	12 (16)	51 (68)	12 (16)			73 (97)	2 (3)	0 (0)			71 (95)	4 (5)	0 (0)	
Fourth Year	9 (13)	46 (66)	15 (22)			70 (100)	0 (0)	0 (0)			67 (96)	3 (4)	0 (0)	
Religion														
Hindus	29 (13.2)	149 (68.1)	41 (18.7)	0.03		219 (100)	0 (0)	0 (0)	<0.001		210 (95.8)	9 (4.2)	0 (0)	0.06
Muslims	5 (10.8)	27 (58.7)	14 (30)			46 (100)	0 (0)	0 (0)			46 (100)	0 (0)	0 (0)	
Others	1 (3.1)	19 (57.5)	13 (40)			27 (82)	6 (18)	0 (0)			30 (91)	3 (9)	0 (0)	
Total	35 (11.7)	195 (65.5)	68 (22.8)			292 (97.9)	6 (2.1)	0 (0)			286 (95.9)	12 (4.1)	0 (0)	

The majority of the study population reported low levels of positive organ donation practice (n=286, 95.9%), which did not reveal any significant difference based on gender, year of study or religion (Table 2).

The mean knowledge and mean practice scores did not show any significant difference based on gender and year of study. Hindus and Muslims had significantly (p<0.001) lower mean knowledge and mean practice scores compared to people following other religion (Table 3).

**Table 3:** Comparison of mean±SD scores of knowledge, attitude, and practice based on gender, year of study, and religion

Variables	Knowledge	Attitude	Practice
Gender			
Males	8.03±1.58	1.76±1.32	0.91±0.57
Females	8.22±1.51	1.37±.0.8	0.91±0.48
p value	0.38	<0.001	0.98
Year of study			
First year	8.08±1.56	1.28±0.78	0.92±0.47
Second year	8.55±1.56	1.43±1.14	1.02±0.44
Third year	8.06±1.21	1.73±1.17	0.86±0.54
Fourth year	8.08±1.30	1.38±0.59	0.86±0.51
p value	0.16	0.02	0.18
Religion			
Hindus	8.00±1.50	0.09±0.00	0.91±0.50
Muslims	8.56±1.53	1.99±0.00	0.78±0.40
Others	8.90±1.42	3.72 ± 1.20	1.15±0.50
p value	<0.001	<0.001	<0.001

The gender difference was seen with males having a significantly (p<0.001) more positive mean±SD attitude (1.76±1.32) than females (1.37±0.8). Furthermore, based on year of study, third-year students showed a significantly (p=0.02) higher mean±SD attitude score (1.37±1.17) than other participants. Hindus and Muslims had a significantly (p<0.001) poorer mean±SD scores (3.72±1.2) compared to those following other religions (Table 3).

Knowledge, attitude and practice had significant positive correlations with each other.

## DISCUSSION

Organ transplantation is the most preferred treatment modality for end-stage organ disease and organ failure. It offers a better quality of life with a better survival benefits. Therefore, the magnitude of organ retrieval is extremely important. The success of retrieval is hugely dependent on the levels of knowledge and attitude of the people. Health care professionals play a vital role in imparting positive knowledge towards organ donation among the people. Therefore, the present study was carried out on one such group of health care professionals to assess and compare the knowledge, attitude and practice regarding organ donation among dental students, based on gender, year of study and religion.

In India, there has been an increase in the number of women taking up dentistry [13]. This has been identified in the present study as well, where the majority (n=238, 79.9%) of the study participants were females. 

According to the study by Coad, *et al* [12], on young adults in the UK, 95% of participants were aware of “organ donation” and “organ transplantation” but very few had responded for the questions like “have you ever donated an organ?” (1.7%) and “did you ever receive an organ for transplantation?” (0.8%); these findings were comparable to our findings (98.6%, 3.4% and 2.4%, respectively). 

In our study, we made a step ahead, by comparing variables like gender, year of study and religion with responses related to organ donation. When gender comparison was done for knowledge scores, a higher mean score was observed among females (8.22±1.51) as compared to males (8.03±1.58). Likewise when levels of correct knowledge was taken into account more females (65.5%) had average (50%–75%) levels of knowledge as compared to males (65%). These findings do not concur with the results reported by Marques, *et al* [14], on medical students attending University of Puerto Rico School of Medicine where almost half (49.6%) of the male participants had adequate knowledge (>50%) compared to females (41.9%).

In our study, males had a significantly (p<0.001) higher mean attitude scores than females. This finding contradicts with the studies by Burra, *et al* [10], and Mekahli, *et al* [15], on European medical students where females had higher positive attitude, as they may have more emotional values compared to males. 

When levels of good practice habits were observed among gender, majority of them had very low levels of practice habits, of whom males (93.3%) were comparable to females (96.6%). However, Bardell, *et al* [16], and Saleem, *et al* [17], they concluded that gender had no association with practice habits. 

In the current study, the average knowledge score was higher among first-year students (66.6%) compared to other year of study. This could be due to the great enthusiasm among the first-year students to gain medical knowledge. Two different studies were conducted by Afshar, *et *al [18], on high school students and medical students in Iran showing that both groups had more knowledge (92% and 85%) due to intensified natural science subjects in the field of medicine and transplantation. This could also be the reason why the first-year students had more knowledge than others.

A higher mean positive attitude score was observed among the third-year students (1.73±1.17) compared to other years (p=0.02). This could be attributed to the fact that third-year students attended medical postings (general medicine and general surgery), and thus are more aware of the need for organs. This finding was in keeping with a study conducted by Bardell, *et al* [16], on medical students of Kingston.

When religion was taken into consideration, Hindus (68%) had higher average knowledge scores (50%–75%) (p=0.03) than others. This might be because of Hindu organizations like Jeeyar Integrated Vedic Academy education are in favor of organ donation and transplantation. In contrast, Hindus (8.00±1.50) and Muslims (8.56±1.53) had lower mean knowledge scores than other religions (Christians, Jains, and Atheists) (8.90±1.42) (p<0.001), which was in agreement with the results of the study by Marques, *et al* [14], where higher mean±SD knowledge scores was noted for Catholics (6.32±1.20) and Protestants (6.26±1.11).

The reason for the lower score among Hindus could be because Hindu culture believes strongly in life after death with a continuos cycle of birth and rebirth. On the other hand, Christianity and Jainisum faith (other religions followed in frequency in India) strongly support organ donation and consider it an example of selfless sacrifice. This thought could have attributed to higher mean knowledge, attitude and practice with organ donation among them.

In our study, Hindus and Muslims had low levels of positive attitude and practice habits. This was in contrast with findings of a study conducted by Saleem, *et al* [17], on adult population in Karachi, where Muslims had higher practice habits; this might be because of people much aware of religious edicts and fatwas from Islamic organizations are more in favor of organ donation. However, other studies by Baput, *et al*, and Tam, *et al* [2, 19], on medical post-graduate and nursing students, respectively, reported that there were no association between religion and practice habits. 

In our study, knowledge, attitude and practice had a significant positive correlation with each other. In contrast, a study by Chung, *et al* [11], on medical students of Hong Kong University illustrated a negative correlation between knowledge and both attitude and practice, and a positive correlation between attitude and action.

The primary limitation of our study was that it was questionnaire-based. Secondly, the study was conducted among only one set of health care professionals in a single institution. Therefore, our results cannot be generalized to the community. More studies should be carried out to rectify the decline in organ donation rate. Government organizations should take up the awareness programs to the masses through social media.

## References

[1] Sahi M (2014). Myths and misconceptions and reality on organ donation. Transplantation Research,.

[2] Tam WWS, Suen LKP, Chan HYL (2012). Knowledge, attitude and commitment toward organ donation among nursing students in Hong Kong. Transplant proc.

[3] Ozdag N, Bal C (2001). The nurses knowledge, awareness and acceptance of tissue-organ donation. EDTNA ERCA J.

[4] Cantwell M, Clifford C (2000). English nursing and medical students’ attitudes towards organ donation. J Adv Nurs.

[5] World Health Organisation (WHO) (2009). Global glossary of terms and definitions on donation and transplantation.

[6] Jeevandan cadaveric transplantation programme Offical website - A government of Andhra Pradesh, 2014.

[7] Dardavessis T, Xenohontos P, Haidich AB, Kristi M (2011). Knowledge, attitude and proposals of medical student concerning transplantations in Greece. Int J Prev Med.

[8] Akgun HS, Bilgin N, Tokalak I (2003). Organ donation : A cross sectional survey of the knowledge and personal views of Turkish health care professionals. Transplant proc.

[9] Lima CX, Lima MVB, Cerqueira RG (2010). Organ donation: Cross sectional survey of knowledge and personal views of Brazilian medical students and physicians. Transplant proc.

[10] Burra P, De Bona M, Canova D (2005). Changing attitude to organ donation and transplantation in university students during the years of medical school in Italy. Transplant Proc.

[11] Chung CKY, Ng CWK, Li JYC (2008). Attitudes, knowledge, and actions with regard to organ donation among Hong Kong medical students. Hong Kong Med J.

[12] Coad L, Carter N, Ling J (2013). Attitudes of young adults from the UK towards organ donation and transplantation. Transplantation Research.

[13] Tandon S (2004). Challenges to the Oral Health Work Force in India. J Dent Edu.

[14] Margues-Lespier JM, Ortiz-Vega NM, Sanchez MC (2013). Knowledge of and attitude toward organ donation: A survey of medical students in Puerto Rico. P R Health SciJ.

[15] Mekahli D, Liutkus A, Fargue S (2009). Survey of first year medical students to assess their knowledge and attitudes toward organ transplantation and donation. Transplant Proc.

[16] Bardell T, Hunter DJW, Kent WDT, Jain MK (2003). Do medical students have the knowledge needed to maximize organ donation rates. Can J Surg.

[17] Saleem T, Ishaque S, Habib N (2009). Knowledge, attitude and practices survey on organ donation among a selected adult population of Pakistan. BMC Medical Ethics.

[18] Afshar R, Sanavi S, Rajabi MR (2012). Attitude and willingness of high school students toward organ donation. Saudi J Kidney Dis Transpl.

[19] Bapat U, Kedlaya PG (2010). Organ donation, awareness, attitudes and beliefs among post graduate medical students. Saudi J Kidney Dis Transpl.

